# Insomnia in Sweden: A Population-Based Survey

**DOI:** 10.1155/2014/843126

**Published:** 2014-05-12

**Authors:** Lena Mallon, Jan-Erik Broman, Torbjörn Åkerstedt, Jerker Hetta

**Affiliations:** ^1^Department of Neuroscience, Psychiatry, Uppsala University, SE-75185 Uppsala, Sweden; ^2^Stress Research Institute, Stockholm University, SE-10691 Stockholm, Sweden; ^3^Department of Clinical Neuroscience, Psychiatry, Karolinska Institutet, SE-14186 Stockholm, Sweden

## Abstract

*Aims*. Estimate the prevalence of insomnia and examine effects of sex, age, health problems, sleep duration, need for treatment, and usage of sleep medication. *Methods*. A sample of 1,550 subjects aged 18–84 years was selected for a telephone interview. The interview was completed by 1,128 subjects (72.8%). *Results*. 24.6% reported insomnia symptoms. Insomnia disorder, that is, insomnia symptoms and daytime consequences, was reported by 10.5%. The prevalence was similar among all age groups, with the exception of women aged 40–49 years who demonstrated a significantly higher prevalence, 21.6%. Having at least one physical or psychiatric disorder was reported by 82.8% of subjects with insomnia disorder. Mean sleep duration for subjects with insomnia disorder was 5.77 hours on weeknights and 7.03 hours on days off/weekends. The corresponding figures for subjects without insomnia disorder were 7.04 hours and 7.86 hours, respectively. Among those with insomnia disorder 62.5% expressed a need for treatment, and 20.0% used prescribed sleep medication regularly. *Conclusions*. Insomnia disorder is highly prevalent in the population. There are significant associations between insomnia disorder and physical and psychiatric disorders. A majority of subjects with insomnia disorder expressed a need for treatment, indicating a public health problem.

## 1. Introduction

Several epidemiological studies have been conducted in order to estimate the prevalence of insomnia in the general population. The reported prevalence rates vary considerably, and differences in how insomnia is defined contribute to this variation.

Also, some variability may be explained by differences in how information is obtained, that is, questionnaire or interview surveys.

Some studies report insomnia symptoms, that is, difficulties initiating and/or maintaining sleep, without restrictive criteria, while others include duration, frequency, or severity criteria. Studies without restrictive criteria produce prevalence estimates from 25% to 48% [1], while studies using some form of duration, frequency, or severity gradations report prevalence rates from 9% to 34% [1, 2]. The diagnosis of insomnia disorder according to classification systems, such as the DSM-IV [3], ICD-10 [4], or ICSD-2 [5] also requires daytime impairment, and with this definition the prevalence rates of insomnia decrease to about 6% to 12% [6, 7]. A consistent finding is that women are more likely to have insomnia [8]. Almost all studies report an increase in insomnia symptoms with age [9, 10], while there are mixed results concerning insomnia disorder [6, 7].

Subjects with insomnia generally report shorter sleep duration compared to normal sleepers [6], and high levels of comorbidity between insomnia and medical disorders have been demonstrated in epidemiological surveys [11, 12]. Among physical disorders heart disease, hypertension, breathing problems, urinary problems, and pain conditions are more common in subjects with insomnia. Moreover, a survey reported that about half of subjects with insomnia also met criteria for a psychiatric disorder [13]. Although effective behavioural and pharmacological therapies exist for insomnia, many insomnia sufferers do not seek help [14] or use sleep medication [6, 14].

It is important to have accurate estimates of the sex- and age-related prevalence of insomnia and its correlates in the general population in order to understand the public health effect of the disorder. Given the differences in the definition of insomnia in epidemiological research it is difficult to draw conclusions about the true prevalence of the disorder.

This study aims to evaluate the prevalence of insomnia and to identify factors associated with insomnia in the general adult population in Sweden. We used a definition of insomnia disorder that includes insomnia symptoms and detrimental effects on daytime functioning, and our definition of insomnia disorder is thereby close to the DSM-IV insomnia disorder diagnosis [3].

## 2. Methods

### 2.1. Procedure

The study was initiated by the Swedish Council on Health Technology Assessment (SBU). Data were collected by a telephone interview commissioned to the Central Bureau of Statistics (SCB), a governmental agency, in Sweden. Data collection was done with a software package designed specifically for this type of computer-assisted phone survey. After a brief description of the aims of the study and after obtaining verbal consent to proceed with the interview, data were collected.

### 2.2. Participants

A sample of 1,550 subjects living in Sweden, 18–84 years of age, representative for the population and proportionally stratified for age and sex was selected for a telephone interview. The interview was completed by 1128 subjects (72.8%). The characteristics of the sample are shown in Table 1. The sample consisted of 52.1% women and the mean age was 47.8 years (SD = 18.0).

### 2.3. Material

The interview consisted of 39 questions covering demographics, work conditions, sleep complaints, daytime impairment due to sleep complaints, sleep duration, physical and psychiatric disorders, need for treatment, and usage of prescriptive sleep medication.

Sleep initiation problems were assessed by asking “How often have you had difficulties falling asleep during the last month?” to be answered on a five-point scale (1 = never or less than once a month; 2 = less than once a week; 3 = 1-2 times per week; 4 = 3–5 times per week; 5 = daily or almost daily). Sleep maintenance problems were assessed by asking “How many times do you wake up during the night?” to be answered on a five-point scale (1 = never; 2 = once; 3 = twice; 4 = 3-4 times; 5 = at least 5 times). Insomnia symptoms were defined as sleep initiation problems at least 3 times per week (scores 4 and 5) and/or sleep maintenance problems at least 3 times per night (scores 4 and 5).

Daytime consequences were assessed by asking “Have your sleep complaints interfered with your daily life during the last month?” (1 = no interference, 2 = minor interference; 3 = moderate interference; 4 = severe interference; 5 = very severe interference).

Insomnia disorder was defined as having insomnia symptoms and at least moderate interference with daytime functioning (scores 3 to 5).

Sleep duration was assessed by asking subjects to estimate sleep duration on weeknights and on days off/weekends. The answer was expressed as a continuous variable. Physical and psychiatric disorders were ascertained by asking (in a yes/no question) if respondents had hypertension, asthma, heart disease, diabetes, gastrointestinal disorder, urogenital disorder, cancer, joint pain, fibromyalgia, psychiatric disorder, burnout, depression, or any other disorder.

Subjects were asked (in a yes/no question) “Do you think you need treatment for your sleep problems?” Usage of prescriptive sleep medication was ascertained by the question “How often during the last month have you used prescriptive sleep medication?” (1 = never or less than once per month, 2 = less than once per week, 3 = 1-2 times per week, 4 = 3–5 times per week, and 5 = daily or almost daily). Usage at least 3 times per week (scores 4 and 5) was considered regular usage. Unfortunately we do not have any information about the nature of sleep medication. The type of sleep medication asked about was “on prescription,” that is, not sold over the counter. In Sweden the dominating drugs in this class are zopiclone, zolpidem, and propiomazine.

### 2.4. Data Analyses

All analyses were carried out using IBM SPSS Statistics, version 20.0. Standard methods were used to calculate mean values and standard deviations (SDs).

When the comparison involved continuous variables the Mann-Whitney *U* test was used, and the chi-square test was used for differences between proportions.

Ninety-five percent confidence intervals were calculated for prevalence rates and odds ratios. Linear regression models were used to calculate correlations between continuous variables. To identify associations between insomnia disorder and physical and mental disorders age-adjusted and multivariate logistic regression analyses were conducted. Results are presented as odds ratios (OR) with 95% confidence intervals.

## 3. Results

### 3.1. Prevalence of Insomnia Symptoms

The prevalence of having difficulties initiating sleep only was 10.7%, and the prevalence of having sleep maintenance problems only was also 10.7%. The prevalence of insomnia symptoms, that is, sleep initiation problems and/or sleep maintenance problems, was 24.6%. Women reported insomnia symptoms more often than men, 29.3% versus 19.4% (*χ* ^2^ = 14.9; *P* < .001). In all age groups insomnia symptoms were more frequent in women (Table 2). Insomnia symptoms were significantly predicted by sex (OR, 1.69; 95% CI, 1.28–2.23; *P* < .001). The highest prevalence of insomnia symptoms was in the oldest age group, 70 to 84 years, in which 31.1% of men and 36.3% of women reported insomnia symptoms. With the youngest age group as a reference we found that insomnia symptoms remained comparable between age groups with the exception of men aged 70 to 84 years who had a significant higher prevalence rate (OR, 2.33 95% CI, 1.12–4.85; *P* < .05).

### 3.2. Prevalence of Insomnia Disorder

Of those with insomnia symptoms 42.9% reported concomitant impairment of daytime functioning and were classified as having insomnia disorder. The prevalence of insomnia disorder was 10.5%, and more women than men reported insomnia disorder, 13.6% versus 7.1% (*χ* ^2^ = 12.7; *P* < .001). In all age groups insomnia disorder was more frequent in women (Table 2). Thus, insomnia disorder was significantly predicted by sex (OR, 2.08; 95% CI, 1.38–3.12; *P* < .001). The prevalence of insomnia disorder remained comparable between age groups with the exception of women aged 40 to 49 years who demonstrated a significant higher prevalence rate, 21.6% (OR, 2.65; 95% CI, 1.20–5.85; *P* < .05).

### 3.3. Sleep Duration

Subjects with insomnia disorder reported shorter sleep duration on weeknights compared to subjects without insomnia disorder, 5.77 ± 1.64 hours versus 7.03 ± 1.05 hours (*t* = 11.2; *P* < .0001). They also reported shorter sleep duration on days off/weekends, 7.04 ± 2.41 hours, compared to subjects without insomnia disorder, 7.86 ± 1.37 hours (*t* = 15.9; *P* < .0001) (Table 3). Both groups extended their sleep on days off/weekends. On days off/weekends 42.4% of subjects with insomnia disorder and 43.4% of subjects without insomnia disorder extended their sleep with at least one hour.

### 3.4. Comorbid Disorders

Having at least one physical or psychiatric disorder was reported by 82.8% of subjects with insomnia disorder compared to 54.2% of subjects without insomnia disorder (OR, 1.78; 95% CI, 1.58–1.98; *P* < .001). In subjects with insomnia disorder there was no sex difference in the prevalence of comorbid disorders (OR, 0.74; 95% CI, 0.24–2.24; n.s.), but there was a difference between age groups (OR, 1.72; 95% CI, 1.20–2.48; *P* < .01). The prevalence of comorbid disorders increased with advancing age, and all of the insomniacs aged 60 and above reported at least one physical or psychiatric disorder. All disorders, with the exception of diabetes and cancer, were more common in subjects with insomnia disorder (Table 4). In unadjusted logistic regression analyses significant associations were found between insomnia disorder and several of the disorders. In multivariate logistic regression analysis including all disorders, the association between insomnia disorder and many of the disorders were reduced to a nonsignificant level. Significant associations remained between insomnia disorder and asthma, gastrointestinal disorders, joint pain, fibromyalgia, and depression. The strongest association found was with depression (OR, 4.91; 95% CI, 2.63–9.17; *P* < .001). In subjects with insomnia disorder there was no sex difference (OR, 0.78; 95% CI, 0.36–1.71; n.s.), or difference between age groups (OR, 0.97; 95% CI, 0.77–1.21; n.s.) in the prevalence of depression.

### 3.5. Need for Treatment and Usage of Prescriptive Sleep Medication

Among those with insomnia disorder 62.5% expressed a need for treatment, and 22.0% used prescriptive sleep medication regularly. There was no age or sex difference in expressed need for treatment or usage of sleep medication. Subjects with insomnia disorder who were depressed used sleep medication more often than insomniacs without depression, 33.3% versus 17.1% (OR, 3.00; 95% CI, 1.22–7.37; *P* < .05).

## 4. Discussion

The main finding of the present study is that the prevalence of insomnia disorder is 10.5%, and women are 2.08 more likely to report insomnia disorder. Insomnia disorder did not increase with advancing age since being older decreased the probability of daytime impairment due to insomnia symptoms. There was, however, a significant rise in reports of insomnia disorder in women aged 40–49 years. Subjects with insomnia disorder slept less than 6 hours on weeknights, but 42.4% were able to extend sleep with more than 1 hour on days off/weekends. There was a strong overlap between insomnia disorder and physical and psychiatric disorders, most often with depression. A majority of subjects with insomnia disorder expressed a need for treatment, indicating that they are troubled and worried about their sleep, while 20.0% used sleep medication.

Advantages of the present study include the nationally representative sample, the high response rate, the broad age range, the comprehensive definition of insomnia disorder, and the wide range physical and psychiatric disorders included. Data were collected by telephone interview, and telephone interviews assessing DSM-IV psychiatric disorders have been shown to yield results comparable to other strategies [15].

One limitation is that sleep maintenance problems were assessed by the number of awakenings, and an additional question about duration of wake time would have been good. However, we think that 3 or more awakenings will include most individuals with sleep maintenance problems. This categorization has been shown to be related to daytime symptoms [16]. Individuals with 1or 2 long awakenings should of course be regarded as having problems, but when we examined sleep duration in this group there were only few subjects with sleep duration less than 6 hours.

The measure of physical and psychiatric health according to number of disorders reported is relatively coarse but common in epidemiologic studies. The reliability of some self-reported diagnoses, for example, diabetes, is good [16], but others have shown poor reliability [17]. In the present study occurrence of depression was based on subjects' response to a single question about having depression. Previously the value of single-item depression screening has been established [18]. A limitation is that certain physical and psychiatric disorders, life style factors such as excessive drinking, and other concomitant medication than sleep-promoting medication which we did not deal with in this study could influence the results. Another restraint is that the question about sleep medication usage focused on prescribed medications.

This study demonstrates that subjects with insomnia disorder had a higher prevalence of several physical and psychiatric disorders compared to subjects without insomnia disorder. The cross-sectional nature of the study does not permit us to disentangle cause and effect; we can only demonstrate associations. Insomnia disorder and other health problems are either causally related to each other or other factors may influence this relationship. As such, we cannot say that insomnia disorder is a result of a physical or psychiatric disorder or that insomnia caused or exacerbated the disorder. However, from longitudinal studies we know that insomnia disorder actually plays a role in disorder development [19–22].

One aim of the present study was to provide valid estimates of the prevalence of insomnia disorder. Our definition of insomnia disorder is based on insomnia symptoms with frequency criteria, accompanying daytime consequences and a duration criterion of four weeks, and is thereby close to the DSM-IV insomnia disorder diagnosis [3]. The prevalence of insomnia disorder in our study was 10.5% and is similar to that of other studies [7, 14, 23].

In this study insomnia disorder was more common in women, but we found no association with advancing age. There was, however, a significant rise in insomnia disorder in women aged 40 to 49 years. Other surveys have also demonstrated a rise in insomnia disorder frequency in middle rather than old age [12, 24].

It has been shown that women more easily express emotional distress and somatic symptoms, like sleep complaints, compared to men [25]. It has also been shown that there are gender differences in coping styles [26] and exposure to stressful life events [27]. Menopause has been suggested as an explanation for the discrepancy between men and women in the prevalence of insomnia in mid-life. A recent study showed that postmenopausal women (53–58 yrs) had more nocturnal awakenings compared to premenopausal women (44–48 yrs), but the frequency of difficulty falling asleep, snoring, and use of sleep medication did not differ between the groups [28].

Although the elderly have difficulties initiating and maintaining sleep many do not report daytime impairment. With advancing age sleep becomes more fragmented and “lighter” due to increased percentage of stage one sleep and decreased percentage of slow wave sleep [29]. It has been shown that there is an age-related reduction in sleep duration and depth required to maintain daytime alertness [30] and that reduced night-time sleep quality in older people does not cause increased daytime sleep propensity [31]. These changes appear to be, at least in part, related to an age-related reduction in the homeostatic drive for sleep and a reduced strength of the circadian signal [32]. Furthermore, a study showed that age was not predictive of insomnia when social satisfaction and activity status were controlled [33], suggesting that life style changes and inactivity accompanying old age can contribute to the lack of reports of daytime impairment.

Generally epidemiological surveys only assess sleep duration without comparing sleep on weeknights to sleep on days off/weekends. In agreement with previous studies subjects with insomnia disorder reported shorter sleep duration than subjects without insomnia disorder [6]. Surprisingly, we found that 42.4% of subjects with insomnia disorder increased their sleep duration with at least one hour on days off/weekends. This may partly be explained by a reduced stress experienced during weekends. Still it casts some doubts about these subjects having true insomnia disorder. This aspect has not been studied extensively earlier but should indeed be investigated in future studies. Our findings run counter to a survey from Spain demonstrating that subjects with insomnia did not to extend their time in bed on weekends [6] but is in agreement with a study from South Korea where subjects with difficulties initiating sleep slept at least three extra hours on days off and weekends [34].

Our study confirms that insomnia disorder rarely occurs alone. It is by far more common as a comorbid condition than as a single sleep problem. We found that 82.8% of subjects with insomnia disorder reported one or more disorders which is similar to findings from a survey where 86.1% of subjects with insomnia reported medical health problems [11]. Depression was reported by 40.5% of subjects with insomnia disorder, and subjects with insomnia disorder were 4.91 times as likely to have depression compared to subjects without insomnia disorder. We found no variation in association between insomnia disorder and depression between age groups, indicating that depression and insomnia disorder are related to each other with similar strength across life. In addition, we found that fibromyalgia, gastrointestinal disorder, and asthma were associated with insomnia disorder. These findings are in line with a survey that demonstrated that insomnia disorder was most strongly associated with psychiatric conditions, conditions characterised by psychological properties, and pain conditions [9]. Also, an association between gastroesophageal reflux disease and sleep problems has previously been reported [35].

In the present study a majority of subjects with insomnia disorder expressed a need for treatment, while 20.0% used prescribed sleep medication regularly. Other surveys from different countries report that a majority of subjects with insomnia do not use sleep medication. Rates of sleep medication usage in subjects with insomnia range from 21.5% to 33.2% [6, 14]. Variation in usage may be due to different definitions of sleep medication usage, but also cultural, social, or economic dissimilarities as well as differences in attitudes to sleep complaints and sleep medication.

## 5. Conclusions

This study provides important information about several aspects of the epidemiology of insomnia disorder, and the results have public health implications. The prevalence of insomnia disorder was 10.5%, and it did not increase with advancing age. This may suggest that many elderly adapt their life stylein such a way that potential daytime consequences of disturbed sleep are not manifested. Insomnia disorder was strongly related to physical and psychiatric disorders, most notably depression, underlining the importance of investigating and treating insomnia in subjects with physical and psychiatric disorders. The cause-effect relationship is difficult to establish, but there is evidence that treatment of insomnia disorder should be considered separately and independent of other cooccurring disorders [36]. A majority of subjects with insomnia disorder expressed a need for treatment, but only one-fifth used prescribed sleep medication. This study confirms that insomnia disorder is a public health issue, and improved recognition and adequate treatment strategies are required.

## Figures and Tables

**Table 1:** Characteristics of the sample (*N* = 1128).

	Men		Women	
	*N*	%	*N*	%
Age groups (years)				
18–29	117	21.7	106	18.0
30–39	102	18.9	89	15.1
40–49	87	16.1	111	18.9
50–59	78	14.4	90	15.3
60–69	95	17.6	101	17.2
70–84	61	11.3	91	15.5
Physical and psychiatric disorders				
Hypertension	68	12.6	107	18.2
Asthma	68	12.6	73	12.4
Heart disease	37	6.9	39	6.6
Diabetes	35	6.5	27	4.6
Gastrointestinal disorder	86	15.9	134	22.8
Urogenital disorder	37	6.9	36	6.1
Cancer	17	3.1	14	2.4
Joint pain	98	18.1	172	29.3
Fibromyalgia	2	0.4	20	3.4
Other physical disorders	45	8.3	90	15.3
Psychiatric disorder	26	4.8	41	7.0
Burnout	19	3.5	35	6.0
Depression	42	7.8	70	11.9
Sleep duration (hours)				
Weeknights (mean ± SD)	6.87 ± 1.12		6.93 ± 1.24	
Weekends/days off (mean ± SD)	7.85 ± 1.51		7.70 ± 1.55	

**Table 2:** Prevalence (%) and odds ratios (95% confidence interval) of insomnia symptoms and insomnia disorder by age groups in men (*n* = 537) and women (*n* = 587).

	Insomnia symptoms		Insomnia disorder	
	Men	Women	Men	Women
Age groups (yrs)				
18–29	16.2 [10.6–24.0]	23.6 [16.5–32.5]	6.8 [3.5–12.9]	9.4 [5.2–16.5]
30–39	17.6 [11.5–26.2]	32.6 [23.7–42.9]	6.9 [3.5–12.9]	13.5 [7.9–22.1]
40–49	18.4 [11.6–27.8]	26.1 [18.8–35.0]	11.5 [6.4–19.9]	21.6 [15.0–30.2]
50–59	15.4 [9.0–25.0]	27.8 [19.6–37.8]	5.1 [2.0–12.5]	13.3 [7.8–21.9]
60–69	21.1 [14.1–30.3]	30.7 [22.5–40.3]	4.2 [1.6–10.3]	12.9 [7.7–20.8]
70–84	31.1 [20.9–43.6]	36.3 [27.1–46.5]	8.2 [3.5–17.8]	9.9 [5.3–17.7]

Total	19.4 [16.2–22.9]	29.3 [25.8–33.1]	7.1 [5.2–9.6]	13.6 [11.1–16.6]

**Table 3:** Sleep duration on week nights and on weekends/days off in subjects with insomnia disorder (*n* = 118) and in subjects without insomnia disorder (*n* = 1005).

	Sleep duration on week nights (hrs)		Sleep duration on weekends/days off (hrs)	
	Mean	SD	Mean	SD
Subjects with insomnia disorder	5.77	1.64	7.04***	2.41
Subjects without insomnia disorder	7.03	1.05	7.86***	1.37

SD: standard deviation: ***P < .001.

**Table 4:** Prevalence (%) and odds ratios (95% confidence interval) of physical and psychiatric disorders in subjects without insomnia disorder (*n* = 1005) and in subjects with insomnia disorder (*n* = 118).

	Subjects without insomnia disorder	Subjects with insomnia disorder	Subjects with insomnia; univariate analyses^a^	Subjects with insomnia; multivariate analyses^b^
	%	%	OR (95% CI)	OR (95% CI)
Hypertension	14.8	22.4	1.67 (1.04–2.67)	0.98 (0.53–1.83)
Asthma	10.9	25.6	2.81 (1.77–4.44)	1.96 (1.11–3.44)
Heart disease	5.9	14.4	2.69 (1.51–4.80)	1.13 (0.50–2.54)
Diabetes	5.1	8.5	1.75 (0.86–3.54)	1.53 (0.67–3.51)
Gastrointestinal disorder	16.3	45.8	4.32 (2.90–6.44)	2.36 (1.46–3.82)
Urogenital disorder	5.8	12.7	2.38 (1.30–4.34)	1.72 (0.80–3.66)
Cancer	2.8	2.5	0.91 (0.27–3.04)	0.30 (0.07–1.24)
Joint pain	21.3	46.6	3.22 (2.18–4.76)	1.91 (1.16–3.15)
Fibromyalgia	1.1	9.5	9.41 (3.98–22.23)	5.04 (1.88–13.53)
Other physical disorder	11.2	18.6	1.83 (1.10–3.02)	1.16 (0.63–2.13)
Psychiatric disorder	3.7	24.1	8.32 (4.86–14.23)	2.00 (0.91–4.37)
Burnout	2.5	23.7	12.17 (6.81–21.76)	2.19 (0.99–4.85)
Depression	6.2	40.5	10.40 (6.64–16.28)	4.91 (2.63–9.17)

OR: odds ratio; CI: confidence interval findings are significant when CIs do not include 1.00.

^a^Adjusted for age in 5-year strata.

^b^Multivariate analyses adjusted for age in 5-year strata, all physical and psychiatric disorders.
